# A Ratiometric Organic Fluorescent Nanogel Thermometer for Highly Sensitive Temperature Sensing

**DOI:** 10.3390/bios12090702

**Published:** 2022-09-01

**Authors:** Chao Wang, Xianhao Zhao, Kaiyu Wu, Shuyi Lv, Chunlei Zhu

**Affiliations:** Key Laboratory of Functional Polymer Materials of Ministry of Education, State Key Laboratory of Medicinal Chemical Biology, Institute of Polymer Chemistry, College of Chemistry, Nankai University, Tianjin 300071, China

**Keywords:** aggregation-induced emission, twist intramolecular charge transfer, thermoresponsive polymer, nanogel, ratiometric sensing, nanothermometer, bactericidal temperature

## Abstract

Sensing temperature in biological systems is of great importance, as it is constructive to understanding various physiological and pathological processes. However, the realization of highly sensitive temperature sensing with organic fluorescent nanothermometers remains challenging. In this study, we report a ratiometric fluorescent nanogel thermometer and study its application in the determination of bactericidal temperature. The nanogel is composed of a polarity-sensitive aggregation-induced emission luminogen with dual emissions, a thermoresponsive polymer with a phase transition function, and an ionic surface with net positive charges. During temperature-induced phase transition, the nanogel exhibits a reversible and sensitive spectral change between a red-emissive state and a blue-emissive state by responding to the hydrophilic-to-hydrophobic change in the local environment. The correlation between the emission intensity ratio of the two states and the external temperature is delicately established, and the maximum relative thermal sensitivities of the optimal nanogel are determined to be 128.42 and 68.39% °C^−1^ in water and a simulated physiological environment, respectively. The nanogel is further applied to indicate the bactericidal temperature in both visual and ratiometric ways, holding great promise in the rapid prediction of photothermal antibacterial effects and other temperature-related biological events.

## 1. Introduction

Temperature is considered as one of the most important physiological parameters for organisms, as it is closely correlated with a variety of biological activities, such as gene expression, enzymatic reactions, and protein synthesis [1,2,3]. In some situations, the subtle variation in temperature also indicates the pathological states of living organisms (e.g., inflammation and tumorigenesis). It is thus of great significance to sensitively sense temperature variation at the nanoscale to explore the thermal behaviors of biological systems [4,5]. Among various types of nanothermometers, the fluorescent ones have attracted extensive attention due to their non-invasiveness, high spatiotemporal resolution, and real-time signal feedback [6,7,8,9]. At present, a number of organic and inorganic fluorescent nanothermometers have been developed [6], including small-molecule organic dyes [10,11], fluorescent proteins [12,13], organic dye-conjugated polymeric nanoparticles [14,15], lanthanide-doped nanoparticles [16,17], quantum dots [18,19], carbon dots [20], and fluorescent nanodiamonds [8]. These nanothermometers typically show rapid responses to external temperature by altering one and/or multiple fluorescence parameters, in which the commonly used ones include the emission intensity, fluorescence lifetime, peak position, emission intensity ratio, and fluorescence polarization anisotropy [6,7,8,9,10,11,12,13,14,15,16,17,18,19,20]. Despite tremendous achievements in the development of fluorescent nanothermometers, it remains challenging to realize highly sensitive temperature sensing due to the limited signal changes during temperature variation. Given the potential of ratiometric methods in amplifying signal differences as well as the good biocompatibility of organic materials, it is preferred to construct ratiometric organic fluorescent nanothermometers for highly sensitive temperature sensing in biological systems.

Thermoresponsive nanogels are fabricated from thermoresponsive polymers (e.g., poly(*N*-isopropylacrylamide) (PNIPAM)) with a crosslinked network, which can exhibit remarkable changes in the local polarity during phase transition [21,22,23,24,25,26]. In terms of the polymers with a lower critical solution temperature (LCST), the hydrated polymeric units at low temperatures will undergo thermally induced phase separation at a temperature higher than LCST due to the entropy loss of bound water surrounding the polymer chains (i.e., dehydration) [21,22,23,24,25,26,27]. During this process, the microenvironment inside the nanogels swifts from a hydrophilic state to a hydrophobic state [21]. Such a unique feature can be employed to regulate the fluorescence properties of polarity-sensitive fluorophores (e.g., nitrobenzoxadiazole [28,29,30]). However, these fluorophores typically exhibit a single-peak change to temperature variation (typically from a non- or weakly emissive state to a highly emissive state), which does not favor the construction of ratiometric nanothermometers to enhance sensing sensitivity. In this aspect, organic fluorophores with characteristic twisted intramolecular charge transfer (TICT) properties are promising to address the aforementioned issue [31,32,33,34,35]. In a non-polar environment, these fluorophores primarily adapt a locally excited (LE) state by showing a blue-shifted and increased emission; in contrast, a polar environment forces them to exhibit a TICT state, which is accompanied by red-shifted and decreased emission [31,32,35]. Since both states coexist within a given environment, it is feasible to combine TICT properties with the phase-transition properties of thermoresponsive polymers to further improve the sensing sensitivity of organic fluorescent nanothermometers.

**Figure 1:**
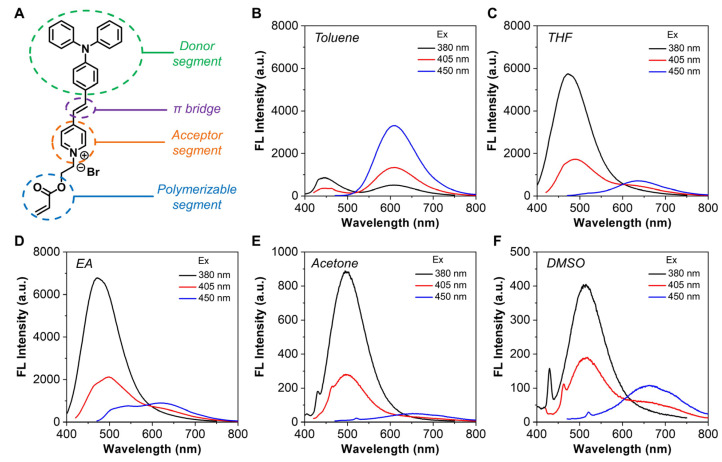
Photophysical characterizations of TVPA. (**A**) Chemical structure of TVPA. (**B**–**F**) Emission spectra of TVPA in (**B**) toluene, (**C**) THF, (**D**) EA, (**E**) acetone, and (**F**) DMSO, respectively. [TVPA] = 10 µM.

Herein, we report a ratiometric fluorescent nanogel thermometer for highly sensitive temperature sensing. The nanogel was composed of a polarity-sensitive aggregation-induced emission (AIE) luminogen TVPA (chemical structure; see Figure 1A) to output dual emissions for ratiometric analysis, a crosslinked thermoresponsive polymer PNIPAM to enable reversible phase transition, and a positively charged surface to provide colloidal stability. When the external temperature varied at a range that covered the LCST of PNIPAM, the dual-emissive AIE luminogen exhibited a reversible change between a blue-emissive state and a red-emissive state. Taking the emission intensity ratio as the variable parameter, it was possible to establish a quantitative correlation with the external temperature for temperature sensing. Notably, the maximum relative thermal sensitivities of the optimized nanogel were determined to be 128.42 and 68.39% °C^−1^ in water and simulated physiological buffer, respectively, which were much higher than most organic fluorescent nanothermometers. As a proof-of-concept demonstration, the resultant nanogel was further applied to sense the bactericidal temperature in both visual and ratiometric ways, which could be used for the rapid prediction of the viability of bacteria after high-temperature treatments.

## 2. Materials and Methods

### 2.1. Chemicals and Materials

Tetrahydrofuran (THF), ethyl acetate (EA), toluene, acetone, sodium chloride (NaCl), and potassium chloride (KCl) were ordered from Tianjin Bohai Chemical Industry Group Co., Ltd. (Tianjin, China). Dimethyl sulfoxide (DMSO) was purchased from Shanghai Adamas Reagent Co., Ltd. (Shanghai, China). NIPAM was purchased from Beijing Ouhe Technology Co., Ltd. (Beijing, China). *N*,*N*′-Methylenebisacrylamide (MBAM) and *N*,*N*,*N*′,*N*′-tetramethylethylenediamine (TEMED) were obtained from Energy Chemical Co., Ltd. (Shanghai, China). Phosphate buffer saline (PBS, 10 mM, pH = 7.4) and ampicillin sodium were ordered from Solarbio Life Science (Beijing, China). Luria-Bertani broth (LB) was purchased from OXOID (Altrincham, England). All chemicals were used as received without further purification. The water used in all experiments was obtained by filtering through a set of HEAL FORCE cartridges (Smart-N15VF, Shanghai, China).

### 2.2. Characterization Methods

All absorption and fluorescence spectra were measured by a UV-vis spectrometer (UV-2600, Shimadzu, Japan) and a fluorescence spectrometer (F-4700, HITACHI, Tokyo, Japan), respectively. The hydrodynamic diameter and zeta potential were measured by dynamic light scattering (DLS) and a zeta potential analyzer (Zetasizer Nano ZS90, Malvern, England). The nanogels were negatively stained with 2 wt.% uranyl acetate prior to examination by transmission electron microscopy (TEM; Tecnai G2 F20, FEI, Hillsboro, QR, USA).

### 2.3. Preparation of Ratiometric Fluorescent Nanogel Thermometers

The polarity-sensitive fluorescent monomer TVPA [36] and the cationic radical initiator 2,2′-azobis-[2-(1,3-dimethyl-4,5-dihydro-1H-imidazol-3-ium-2-yl)]propane triflate (ADIP) [37] were prepared by referring to previously published papers. NIPAM (200 μmol), MBAM (2 μmol), and TEMED (58 μmol, 0.8 μL) were mixed in water (1.9 mL) with different molar ratios of TVPA (NIPAM:TVPA = 100:1, 200:1, 300:1, and 400:1). The mixture was degassed by argon at 70 °C for 10 min to remove dissolved oxygen. Next, ADIP (28 μmol, 16.9 mg) that was pre-dissolved in water (100 μL) was added to the reaction system to initiate polymerization, followed by stirring at 70 °C for 1 h. After cooling to room temperature, the mixture was subjected to salt-induced precipitation (i.e., one-time precipitation with saturated NaCl solution (40 mL)). The crude product was dialyzed against water, followed by freeze-drying to afford pale-yellow powders.

### 2.4. Precipitation of Nanogels with Different Salting-Out Methods

Following the aforementioned fabrication procedure, the nanogel (NIPAM:TVPA = 200:1) was precipitated under different salting-out conditions. (i) The first one was subjected to one-time precipitation using saturated NaCl solution (40 mL). (ii) The second one was subjected to one-time precipitation using over-saturated NaCl solution (40 mL) that was prepared by adding excess NaCl solid into water until over-saturation. (iii) The third one was subjected to stepwise precipitation, which was achieved by pouring the mixture into unsaturated NaCl solution (500 mM, 40 mL), followed by passing it through a 0.8 μm filter. The filtrate was then precipitated by adding excess NaCl solid into water until over-saturation. All collected precipitates were dialyzed against water and freeze-dried for subsequent use. For the convenience of discussion, the nanogels obtained from methods (i), (ii), and (iii) are denoted as NG-1, NG2, and NG3, respectively.

### 2.5. Thermoresponsive Spectral Changes of Nanogels

The temperature-dependent emission spectra of NG-1, NG-2, and NG-3 were measured in water and KCl solutions with different concentrations (100 mM, 150 mM, and 200 mM). To achieve precise temperature control, the fluorometer was attached to a circulating bath with cooling and heating functions. All samples were equilibrated at a given temperature for 10 min prior to the measurement of emission spectra (Ex = 405 nm).

### 2.6. Calculation of Relative Thermal Sensitivity and Temperature Resolution

The relative thermal sensitivity (*S_r_*) was defined as:

(1) $$S_{r\;}=\frac{1}{R}\left|\frac{\partial R}{\partial T}\right|$$

where *S_r_* was presented as the percent change per degree Celsius (i.e., % °C^−1^), and *R* was the emission intensity ratio at two peak positions.

The temperature resolution (*δT*) was defined as:

(2) $$\delta T=\frac{1}{S_{r}}\frac{\delta R}{R}$$

where *δT* was expressed as the degree Celsius (i.e., °C).

### 2.7. Bacterial Culture

The bacterial strain used in this study was ampicillin-resistant *Escherichia coli* (*E. coli*^Ampr^), which was provided by Prof. Qiong Yang (Beijing Normal University). A single colony of *E. coli*^Ampr^ on a solid agar plate was transferred to an LB medium (5 mL) with 100 µg mL^−1^ ampicillin sodium in a shaking incubator (170 rpm) at 37 °C overnight. After culturing overnight, the bacterial cells were harvested by centrifugation (3500× *g*, 5 min) and washed with PBS three times. The supernatant was discarded, and the remaining bacterial cells were resuspended in PBS, which were then diluted to an optical density of 1.0 at 600 nm (OD_600_ = 1.0) for subsequent use.

### 2.8. Sensing the Bactericidal Temperature with Nanogel Thermometers

The bacterial cells (500 µL, OD_600_ = 1.0) were diluted by 2-fold using the PBS solution of NG-3 at a final concentration of 0.5 mg mL^−1^. For comparison, the bacterial suspension diluted with PBS was set as the blank group. The bacterial suspensions were then incubated at different temperatures (30, 40, and 50 °C) for 15 min, followed by recording the emission spectra (Ex = 405 nm). The emission intensity ratio at two peak positions was used for quantitative analysis. Furthermore, the bacterial viabilities post-incubation at different temperatures were evaluated using the plate-counting assay. Specifically, the treated bacterial suspensions were diluted by 1 × 10^3^-fold with PBS, followed by spreading the diluent (100 µL) on LB agar plates with 100 µg mL^−1^ ampicillin sodium for the overnight culture at 37 °C. The colony-forming units (CFU) number of each plate was counted, and the percentage of the CFU number was calculated for quantitative analysis (*n* = 3).

## 3. Results and Discussion

To enable ratiometric temperature sensing, a polarity-sensitive AIE luminogen TVPA was synthesized by referring to a previous report [36]. As shown in Figure 1A, TVPA had a typical donor (D)−π−acceptor (A) structure, with the triphenylamine, vinyl, and pyridinium groups serving as the donor, π bridge, and acceptor, respectively. In addition, an acrylate group was attached to the molecular skeleton to favor radical polymerization. We only performed basic photophysical characterizations on TVPA, as the detailed optical properties have been reported in the previous publication [36]. As shown in Figure S1, TVPA exhibited a predominant absorption in the range of 360–550 nm, with the absorption maximum located at ca. 465 nm. Although the previous report demonstrated that TVPA possessed prominent TICT properties due to the presence of a D−π−A structure, further investigation indicated that the emission profiles of TVPA were highly dependent on the excitation wavelength. As shown in Figure 1B–F, when using 380 nm as the excitation wavelength, the emission maxima of TVPA gradually red-shifted as the solvent polarity increased from toluene to DMSO, which was basically consistent with the previous study [36]. Interestingly, when the excitation wavelength was set at 405 nm, two emission peaks were identified in almost all solvents. We inferred that the short-(400–600 nm) and long-wavelength (550–800 nm) emissions corresponded to the LE (dominant in non-polar environments) and TICT (dominant in polar environments) states, respectively. To verify our hypothesis, the excitation wavelength was further extended to 450 nm; in this situation, only the long-wavelength emission could be observed. Given that the dual-emissive attribute was highly favorable for ratiometric analysis, the excitation wavelength was fixed at 405 nm for subsequent studies.

**Figure 2:**
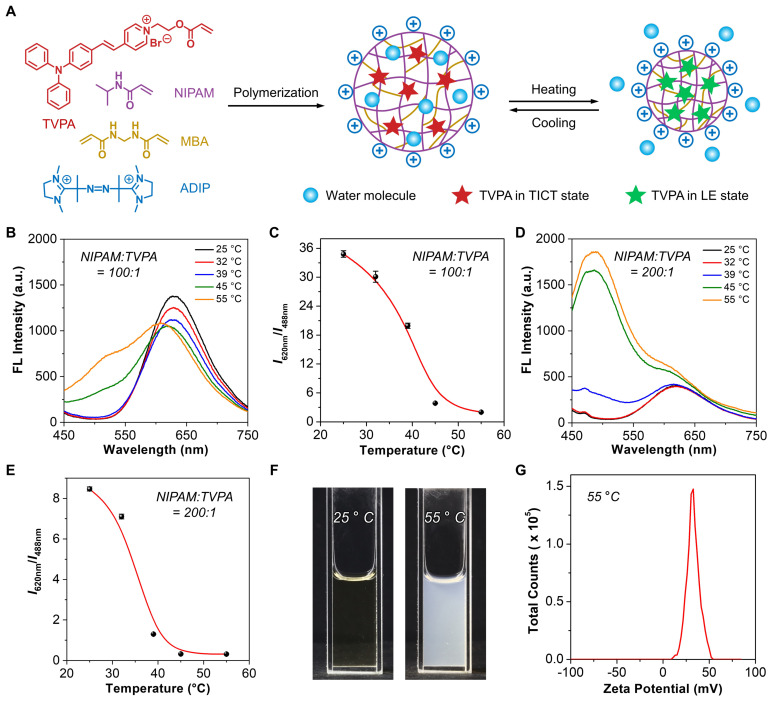
Fabrication of nanogels with different molar ratios of NIPAM and TVPA. (**A**) Schematic illustration showing the polymerization process and thermoresponsive behaviors. (**B**) Temperature-dependent emission spectra of the nanogel (NIPAM:TVPA = 100:1, 0.25 mg mL^−1^). Ex = 405 nm. (**C**) Changes in the ratio of the emission intensities at 620 and 488 nm from panel (**B**) as a function of temperature (*n* = 3). (**D**) Temperature-dependent emission spectra of the nanogel (NIPAM:TVPA = 200:1, 0.25 mg mL^−1^). Ex = 405 nm. (**E**) Changes in the ratio of the emission intensities at 620 and 488 nm from panel (**D**) as a function of temperature (*n* = 3). (**F**) Photographs of the nanogel (NIPAM:TVPA = 200:1, 2 mg mL^−^^1^) at 25 and 55 °C, respectively. (**G**) Plot of the zeta potential of the nanogel (NIPAM:TVPA = 200:1, 0.25 mg mL^−1^) at 55 °C.

The nanogel was then fabricated via radical polymerization, in which TVPA and NIPAM served as the monomers to provide polarity- and temperature-responsiveness, respectively; MBA was used as the crosslinker to form a crosslinked three-dimensional network; and ADIP acted as the initiator to trigger polymerization and stabilize the formed nanogel with a cationic surface (Figure 2A). During the temperature-induced phase transition of PNIPAM, TVPA could exhibit variable dual emissions as a result of the changes in the polarity of its residing environment. To maximize ratiometric signal changes, a set of nanogels with different molar ratios of NIPAM and TVPA (i.e., 100:1, 200:1, 300:1, and 400:1) were fabricated, and the corresponding emission spectra were recorded at different temperatures. As shown in Figure 2B,D and Figure S2A,C, all nanogels exhibited temperature-dependent spectral changes, in which the two major emission peaks located at 450–550 and 600–700 nm showed opposite variations. As the external temperature increased, the short-wavelength emission remarkably intensified, which was accompanied by the gradual attenuation and/or disappearance of the long-wavelength emission. This phenomenon suggested that the local environment surrounding TVPA underwent a hydrophilic-to-hydrophobic change due to the phase transition of PNIPAM, which forced the TVPA to shift from the red-emissive TICT state to the blue-emissive LE state. Furthermore, the emission intensity ratio at the maximum wavelengths was used as the variable parameter to establish a correlation with the external temperature. As shown in Figure 2C,E and Figure S2B,D, all nanogels exhibited significantly decreased emission intensity ratios when the external temperature increased from 25 to 55 °C. By referring to Equations (1) and (2), the relative thermal sensitivity (*S_r_*) and temperature resolution (*δT*) were calculated and summarized in Table S1. Among all nanogels, the one with the molar ratio of 200:1 showed the optimal *S_r_* (50–65% °C^−1^) and comparable *δT* (0.02–0.05 °C) in the range of 32–45 °C, with the maximum relative thermal sensitivity ($S_{\;r}^{\max}$) of 63.60% °C^−1^ at 39 °C. It should be noted that, although the $S_{\;r}^{\max}$ of the nanogel with the molar ratio of 100:1 was 69.80% °C^−1^ at 45 °C, the sensitive sensing range was only limited to 39–45 °C. Given the remarkable spectral changes and the optimal thermometric performance, the nanogel with the molar ratio of 200:1 was selected for subsequent studies. To verify that the unique spectral changes were triggered by the phase transition of PNIPAM, we also recorded the photographs of the nanogel (NIPAM:TVPA = 200:1) at 25 and 55 °C. As shown in Figure 2F, the transparent nanogel solution became turbid when the external temperature increased from 25 to 55 °C, suggesting the dehydration of PNIPAM due to the remarkable phase separation. Since a cationic radical initiator, ADIP, was used for polymerization, the measured zeta potential of the nanogel was +32.6 mM (Figure 2G). According to the established calibration curve, the loading content of TVPA in the nanogel (NIPAM:TVPA = 200:1) was determined to be 0.21 wt.% (Figure S3).

**Figure 3:**
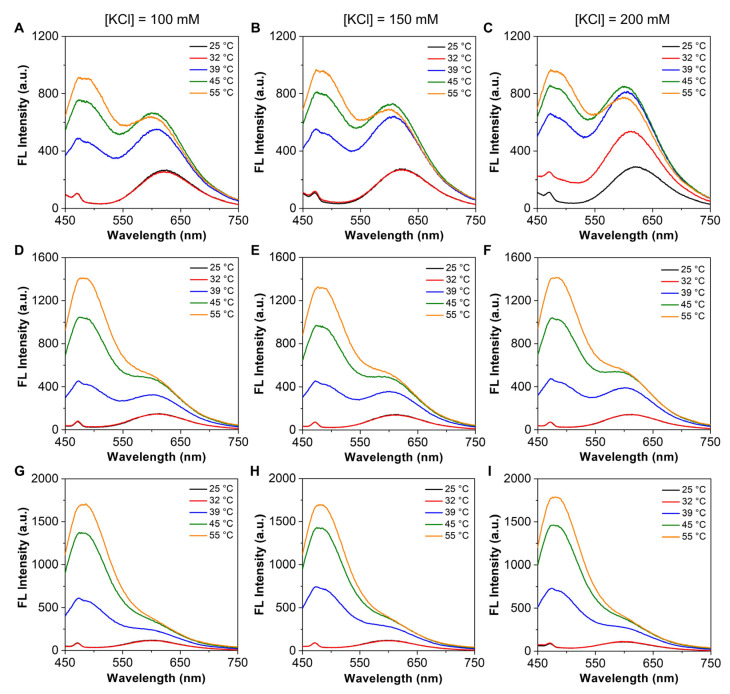
Temperature-dependent changes in the emission spectra of nanogels under different salting-out conditions. (**A**–**C**) Emission spectra of NG-1 (0.25 mg mL^−1^) in (**A**) 100, (**B**) 150, and (**C**) 200 mM KCl solutions at different temperatures. (**D**–**F**) Emission spectra of NG-2 (0.25 mg mL^−1^) in (**D**) 100, (**E**) 150, and (**F**) 200 mM KCl solutions at different temperatures. (**G**–**I**) Emission spectra of NG-3 (0.25 mg mL^−1^) in (**G**) 100, (**H**) 150, and (**I**) 200 mM KCl solutions at different temperatures. Ex = 405 nm.

To ensure the signal stability of nanogels in buffered salt solutions, we further optimized the precipitation process by altering the salting-out conditions. In our study, three salting-out methods were tested to obtain the nanogels, which were one-time precipitation with saturated NaCl solution (denoted as NG-1), one-time precipitation with over-saturated NaCl solution (denoted as NG-2), and preliminary precipitation with unsaturated NaCl solution followed by adding NaCl solid until over-saturation (denoted as NG-3). To compare the anti-interference capabilities of these nanogels, we recorded the temperature-dependent emission spectra in different concentrations of KCl solutions. As shown in Figure 3, no significant changes were found in the emission spectra of NG-2 and NG-3, which were different from those of NG-1. In addition, in contrast to NG-2, the relative spectral changes of NG-3 at long- and short-wavelengths were more complete, with the emission appearing as a single peak at 25 and 55 °C, respectively. Taking the emission intensity ratio as the variable parameter, we further plotted the correlation curve in different concentrations of KCl solutions. As shown in Figure S4, among the three types of nanogels, only NG-3 exhibited the completely overlapped curves, suggesting its robustness and reliability. Further calculation on the *S_r_* and *δT* showed that NG-3 had the highest $S_{\;r}^{\max}$ and the most stable sensing performance in buffered solutions (Tables S2–S4). We inferred that the stepwise salting-out method desirably removed the highly charged nanogels in the first step, leaving the remaining nanogels less susceptible to ionic strength.

**Figure 4:**
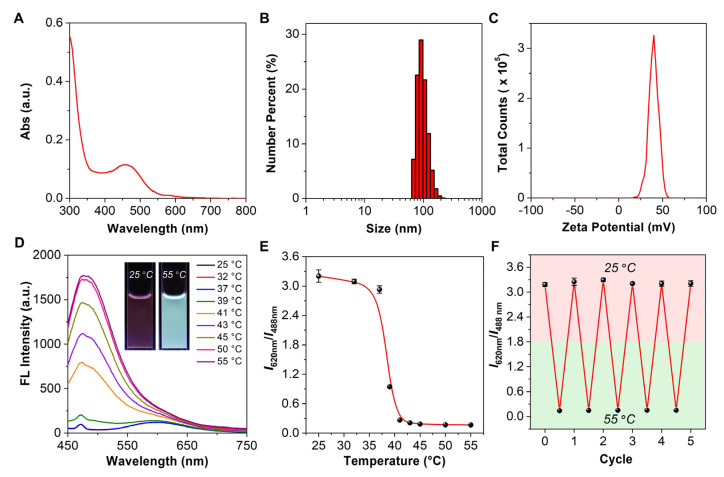
Detailed characterizations of the physiochemical properties of NG-3 in water. (**A**) Absorption spectrum of NG-3. (**B**) Particle size distribution of NG-3 (0.25 mg mL^−1^) at 55 °C. (**C**) Plot of the zeta potential of NG-3 at 55 °C. (**D**) Temperature-dependent emission spectra of NG-3 (0.25 mg mL^−1^) in water. Ex = 405 nm. Inset: fluorescence photographs of NG-3 (0.25 mg mL^−1^) at 25 and 55 °C, respectively, taken under a 365 nm UV lamp. (**E**) Changes in the ratio of the emission intensity at 620 and 488 nm as a function of temperature (*n* = 3). (**F**) Changes in the ratio of the emission intensity at 620 and 488 nm for repeated heating and cooling cycles between 25 and 55 °C (*n* = 3).

In view of the optimal performance of NG-3, we further performed detailed characterizations of its physiochemical properties. The successful introduction of TVPA into the nanogel was confirmed by the UV-vis spectrum of NG-3 (Figure 4A), and the loading content of TVPA in NG-3 was determined to be 0.20 wt.%. The hydrodynamic diameter of NG-3 was distributed from 60 to 110 nm at 55 °C, together with a zeta potential of +39.9 mV (Figure 4B,C). The TEM image showed that the dried NG-3 was spherical in shape, with an average diameter of ca. 25 nm (Figure S5A). Similarly, NG-3 possessed the phase-transition properties at high temperatures (Figure S5B). Furthermore, the thermoresponsive behaviors of NG-3 were evaluated by recording the emission spectra at different temperatures. As shown in Figure 4D, when the external temperature elevated from 25 to 55 °C, the long-wavelength emission gradually disappeared, which was accompanied by the appearance of a new peak at the short-wavelength region. Such a process could also be visualized simply by irradiating the solution of NG-3 under a UV light, in which the emission color changed from dark red at 25 °C to light blue at 55 °C (inset in Figure 4D). The correlation curve with the external temperature was then plotted (Figure 4E), and the corresponding thermometric parameters of NG-3 were calculated (Table 1). In water, the $S_{\;r}^{\max}$ and *δT* at 41 °C were determined to be 128.42% °C^−1^ and 0.01 °C, respectively, which were much higher than most organic fluorescent nanothermometers. In addition, we also tested the emission intensity ratio of NG-3 after repeated heating and cooling cycles between 25 and 55 °C. As shown in Figure 4F, even after five rounds of thermal cycling, there were no noticeable changes in the emission intensity ratio, suggesting the reversibility of NG-3 for repeated uses. Since the spectral changes of NG-3 were barely affected by ionic strength, we also examined the thermoresponsive behaviors of NG-3 in a simulated physiological environment (i.e., 150 mM KCl solution) with added temperature points (Figure S6). The detailed calculation parameters were summarized in Table 1, and the $S_{\;r}^{\max}$ and *δT* at 37 °C were determined to be 68.39% °C^−1^ and 0.02 °C, respectively, suggesting its potential in highly sensitive temperature sensing. In addition, we also evaluated the impact of pH values on the performance of NG-3. As the pH values increased from 5 to 8, there was a slight decrease in the emission intensity ratio (Figure S7). We inferred that the alkaline environment may partially affect the hydration status of the 4,5-dihydroimidazolium group in the surface of the nanogel, leading to the varied emission intensity ratio.

**Figure 5:**
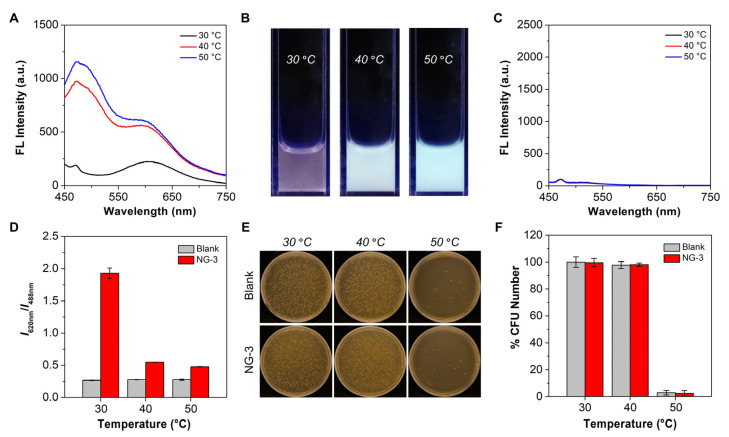
Visual and spectroscopic temperature sensing on bacterial suspensions with NG-3 (0.5 mg mL^−1^). (**A**) Emission spectra of bacteria (*E. coli*^Ampr^) in the presence of NG-3 at different temperatures. Ex = 405 nm. (**B**) Fluorescence photographs of bacteria in the presence of NG-3 at different temperatures taken under a 365 nm UV lamp. (**C**) Emission spectra of bacteria at different temperatures (defined as the blank group). Ex = 405 nm. (**D**) Changes in the ratio of the emission intensity at 620 and 488 nm from panels (**A**,**C**) as a function of temperature (*n* = 3). (**E**) Photographs of bacterial colonies formed on agar plates in the absence and presence of NG-3 at different temperatures. (**F**) Bacterial viability in the absence and presence of NG-3 at different temperatures (*n* = 3).

As a proof-of-concept demonstration, NG-3 was employed as the nanothermometer to sense the bactericidal temperature. Given that the presence of bacteria might affect the temperature sensing capability of NG-3, we first verified its spectral responsiveness post-incubation with *E. coli*^Ampr^ (a drug-resistant Gram-negative bacterial strain) at different temperatures (Figure 5A–C). In contrast to the blank group, when the incubation temperature changed from 30 to 50 °C, NG-3 exhibited sensitive responses in both emission spectra and emission colors. Furthermore, we quantified the intensity ratio of the dual emissions at different incubation temperatures. As shown in Figure 5D, in contrast to the blank group with constant emission intensity ratios, the NG-3 group had distinct specific values at 30, 40, and 50 °C, respectively. It should be noted that the emission intensity ratio was different from that in Figure S6B, as the test conditions were quite different. Since bacterial viability was closely correlated with the temperature of their residing environment, we evaluated the CFU numbers of bacteria post-incubation at different temperatures using the plate-counting assay. As shown in Figure 5E,F, when the incubation temperature was lower than or equal to 40 °C, the bacterial viability was basically unaffected, and the presence of NG-3 did not bring additional toxicity. However, when the incubation temperature was increased to 50 °C, there was a drastic reduction in the bacterial viability, with a killing efficiency of >95%, which was attributed to the hyperthermia-induced protein denaturation and/or membrane disruption. Since photothermal therapy (PTT) is considered as a potent strategy to inactivate drug-resistant bacteria [38,39,40], NG-3 could be employed to visually and spectroscopically reflect the photothermal equilibrium temperature in a real-time fashion, providing a simple and rapid method to foresee the photothermal antibacterial effect.

## 4. Conclusions

In summary, a ratiometric fluorescent nanogel thermometer has been developed for highly sensitive temperature sensing. Three major components were present in the nanogel, including a polarity-sensitive AIE luminogen with dual emissions (i.e., TVPA), a thermoresponsive polymer with phase transition properties (i.e., PNIPAM), and an ionized surface with net positive charges. Upon heating to a temperature beyond the LCST of PNIPAM, the residing environment of TVPA underwent a hydrophilic-to-hydrophobic change, leading to the spectral shift of TVPA from a red-emissive TICT state to a blue-emissive LE state. Taking the emission intensity ratio as the variable parameter, ratiometric temperature sensing was achieved by the optimized nanogel (i.e., NG-3), and the $S_{\;r}^{\max}$ was determined to be 128.42 and 68.39% °C^−1^ in water and a simulated physiological environment, respectively. As a proof-of-concept demonstration, the resultant nanogel was used to indicate the bactericidal temperature in both visual and ratiometric ways, holding great promise in the rapid prediction of photothermal antibacterial effects and other temperature-related biological events.

## Figures and Tables

**Table 1:** Thermometric performance of NG-3 at different temperatures.

*T* (°C)	*S_r_* (% °C^−1^) in Water	*δT* (°C) in Water	*S_r_* (% °C^−1^) in 150 mM KCl	*δT* (°C) in 150 mM KCl
32	0.43	3.63	0.26	11.67
37	1.13	2.31	68.39	0.02
39	105.15	0.01	34.00	0.02
41	128.42	0.01	10.31	0.04
43	13.73	0.09	6.13	0.14
45	6.61	0.15	3.98	0.24
50	1.80	0.64	2.16	0.20
55	0.22	12.08	1.38	1.30

## Data Availability

Not applicable.

## Supplementary Materials

The following supporting information can be downloaded at: https://www.mdpi.com/article/10.3390/bios12090702/s1, Figure S1: Absorption spectrum of TVPA in DMSO; Figure S2: Fabrication of nanogels with different molar ratios of NIPAM and TVPA; Figure S3: Quantification of TVPA in the nanogel (NIPAM:TVPA = 200:1); Figure S4: Changes in the ratio of the emission intensity at 620 and 488 nm as a function of temperature at different concentrations of KCl solutions; Figure S5: Characterizations of the physical properties of NG-3; Figure S6: Changes in the fluorescence signals of NG-3 in 150 mM KCl solution; Figure S7: Impact of pH values on the ratio of the emission intensities of NG-3 at 620 and 488 nm; Table S1: Thermometric performance of nanogels with different molar ratios of NIPAM and TVPA at different temperatures; Table S2: Thermometric performance of NG-1 at different temperatures; Table S3: Thermometric performance of NG-2 at different temperatures; Table S4: Thermometric performance of NG-3 at different temperatures.
