# Supplementary material for: A Ratiometric Organic Fluorescent Nanogel Thermometer for Highly Sensitive Temperature Sensing
Source: Biosensors (Basel). 2022 Sep 1;12(9):702. doi: 10.3390/bios12090702 (PMC9496083; doi:10.3390/bios12090702)
Supplement: Supplementary file 1 [file biosensors-12-00702-s001.zip › biosensors-1865052-supplementary.pdf]

Supplementary Materials

A Ratiometric Organic Fluorescent Nanogel Thermometer for Highly Sensitive Temperature Sensing

Chao Wang [†] **, Xianhao Zhao** [†] **, Kaiyu Wu, Shuyi Lv and Chunlei Zhu***

Key Laboratory of Functional Polymer Materials of Ministry of Education, State Key Laboratory of Medicinal Chemical Biology, Institute of Polymer Chemistry, College of Chemistry, Nankai University, Tianjin 300071, China

*Correspondence: chunlei.zhu@nankai.edu.cn

[†] These authors contributed equally to this work.

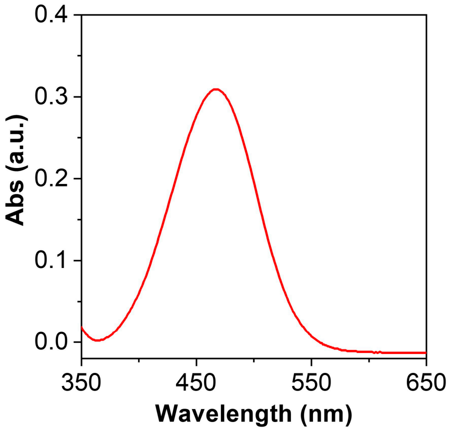

Figure S1. Absorption spectrum of TVPA in DMSO. [TVPA] = 10 μM.

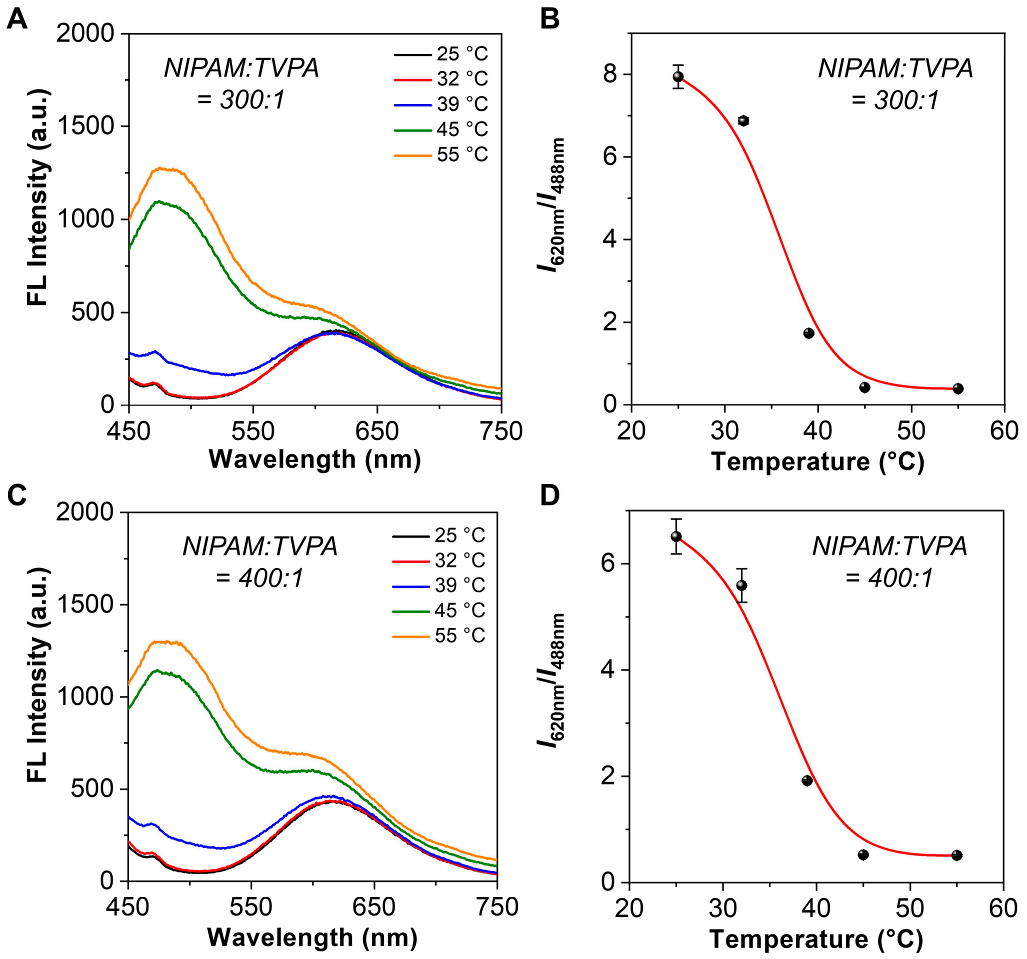

Figure S2. Fabrication of nanogels (0.25 mg mL^{-1}) with different molar ratios of NIPAM and TVPA. (**A**) Temperature-dependent emission spectra of the nanogel (NIPAM:TVPA = 300:1). Ex = 405 nm. (**B**) Changes in the ratio of the emission intensities at 620 and 488 nm from panel (A) as a function of temperature (n = 3). (**C**) Temperature-dependent emission spectra of the nanogel (NIPAM:TVPA = 400:1). (D) Changes in the ratio of the emission intensities at 620 and 488 nm from panel (**C**) as a function of temperature (n = 3). Ex = 405 nm.

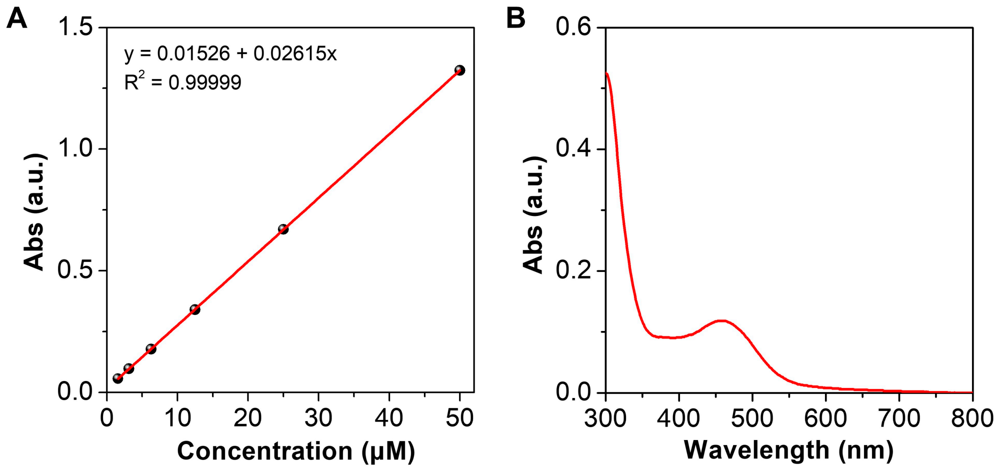

Figure S3. Quantification of TVPA in the nanogel (NIPAM:TVPA = 200:1). (**A**) Calibration curve of TVPA in water. (**B**) Absorption spectrum of NG-1 (2 mg mL^{-1}) in water.

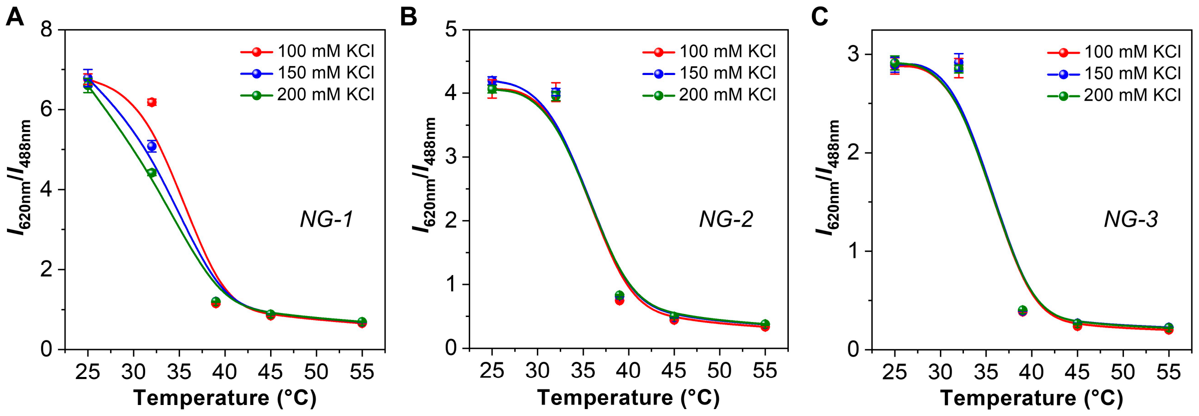

Figure S4. Changes in the ratio of the emission intensity at 620 and 488 nm as a function of temperature at different concentrations of KCl solutions (n = 3). (**A**) NG-1. (**B**) NG-2. (**C**) NG-3.

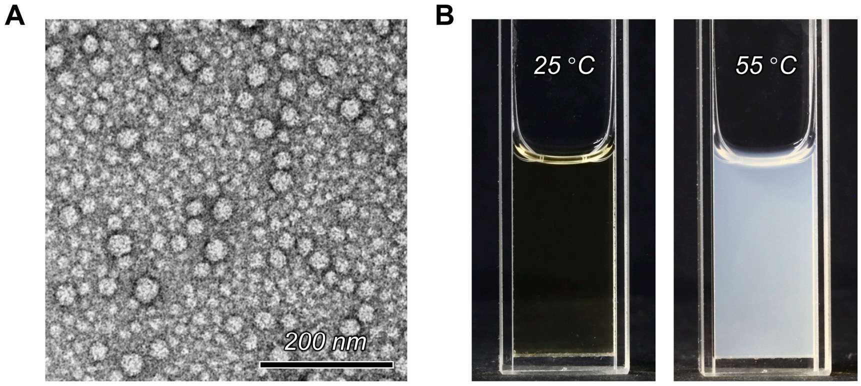

Figure S5. Characterizations of the physical properties of NG-3. (A) TEM image of NG-3. (B) Photographs of NG-3 (2 mg mL^{-1}) in water at 25 and 55 °C, respectively.

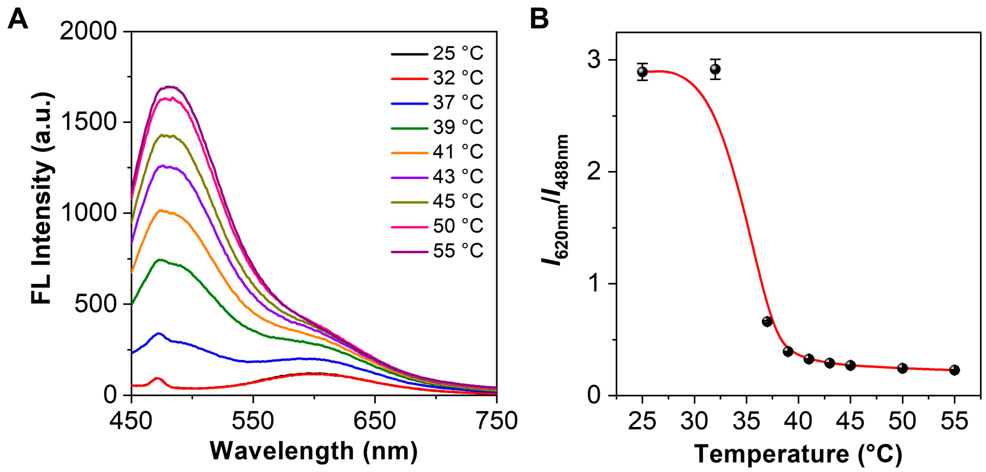

Figure S6. Changes in the fluorescence signals of NG-3 (0.25 mg mL^{-1}) in 150 mM KCl solution. (**A**) Emission spectra of NG-3 at different temperatures in the presence of 150 mM KCl solution. Ex = 405 nm. (**B**) Changes in the ratio of the emission intensity at 620 and 488 nm from panel (**A**) as a function of temperature (n = 3).

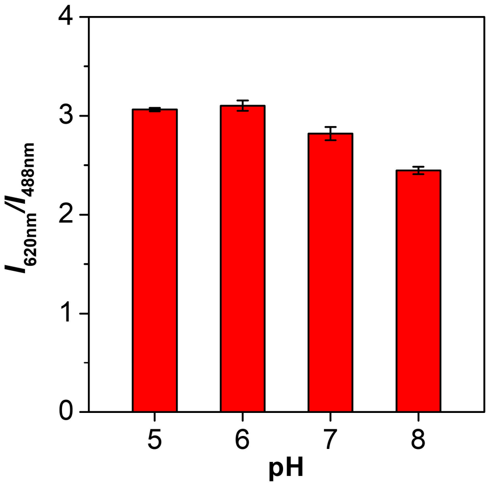

Figure S7. Impact of pH values on the ratio of the emission intensities of NG-3 at 620 and 488 nm.

Table S1. Thermometric performance of nanogels with different molar ratios of NIPAM and TVPA at different temperatures.

T (°C)	**100:1**		**200:1**		**300:1**		**400:1**	
	S_r (% °C^{-1})	δT (°C)	S_r (% °C^{-1})	δT (°C)	S_r (% °C^{-1})	δT (°C)	S_r (% °C^{-1})	δT (°C)
32	2.25	1.69	2.74	0.61	2.22	0.40	2.36	2.05
39	7.27	0.37	63.60	0.02	42.41	0.02	27.36	0.01
45	69.80	0.02	50.60	0.05	51.74	0.04	44.56	0.05
55	9.34	0.10	0.24	14.23	0.70	10.85	0.21	14.44

Table S2. Thermometric performance of NG-1 at different temperatures.

T **(°C)**	**100 mM KCl**		**150 mM KCl**		**200 mM KCl**	
	S_r (% °C^{-1})	δT (°C)	S_r (% °C^{-1})	δT (°C)	S_r (% °C^{-1})	δT (°C)
32	0.81	0.44	4.43	1.04	18.39	0.95
39	61.98	0.02	45.91	0.01	16.54	0.02
45	6.16	0.03	5.96	0.03	4.66	0.03
55	2.75	8.27	2.68	9.43	2.50	8.83

Table S3. Thermometric performance of NG-2 at different temperatures.

T (°C)	100 mM KCl		150 mM KCl		200 mM KCl	
	S_r (% °C^{-1})	δT (°C)	S_r (% °C^{-1})	δT (°C)	S_r (% °C^{-1})	δT (°C)
32	0.21	1.34	0.65	0.52	0.39	0.65
39	62.40	0.01	56.06	0.01	53.62	0.02
45	11.43	0.01	11.42	0.01	11.19	0.01
55	3.18	2.04	2.96	2.59	3.13	8.02

Table S4. Thermometric performance of NG-3 at different temperatures.

T (°C)	100 mM KCl		150 mM KCl		200 mM KCl	
	S_r (% °C^{-1})	δT (°C)	S_r (% °C^{-1})	δT (°C)	S_r (% °C^{-1})	δT (°C)
32	0.13	1.24	0.26	1.11	0.37	0.51
39	91.56	0.02	91.75	0.01	86.96	0.01
45	10.21	0.01	7.69	0.02	8.67	0.02
55	1.83	1.39	1.84	7.66	1.87	3.76
